# The Response of *Oxytropis aciphylla* Ledeb. Leaf Interface to Water and Light in Gravel Deserts

**DOI:** 10.3390/plants12233922

**Published:** 2023-11-21

**Authors:** Zhanlin Bei, Xin Zhang, Fang Zhang, Xingfu Yan

**Affiliations:** 1 School of Biological Science and Engineering, North Minzu University, Yinchuan 750021, China; realpal00147@163.com (Z.B.); zhangfang20022023@163.com (F.Z.); xxffyan@126.com (X.Y.); 2 Key Laboratory of Ecological Protection of Agro-Pastoral Ecotones in the Yellow River Basin, National Ethnic Affairs Commission of the People’s Republic of China, Yinchuan 750021, China

**Keywords:** Gobi, *Oxytropis aciphylla* Ledeb., Trichomes, water collection, light reflection

## Abstract

In arid areas, the scarcity of rainfall severely limits the growth of plants in the area. In arid sandy deserts, plants survive by deeply rooting to absorb groundwater. In arid gravel soil deserts (Gobi), the gravel in the soil layer limits the growth and water absorption of local plant roots. Therefore, the strategies adopted by local plants to obtain water to sustain life have become crucial. *Oxytropis aciphylla* Ledeb. is a perennial, strongly xerophytic, cushion-shaped semi-shrub plant widely distributed in arid gravel desert areas. Its plant height is relatively short, its crown width is not large, and its root system is also underdeveloped. There are small and curly pinnate compound leaves and dense hairy fibers on the surface of the leaves. In this study, we focused on the function of leaf surface trichomes by observing the leaf submicroscopic structure, conducting in situ water harvesting experiments, measuring reflectance spectra, and analyzing chloroplast genomes of *O. aciphylla* leaves. The experimental results indicate that the surface of the leaves of *O. aciphylla* is densely covered with hair-like fiber arrays, and these hair-like fiber surfaces have micro and nanoscale protrusions. These structures can quickly capture moisture in the air and filter out ultraviolet and infrared rays from the sun, without affecting the normal photosynthesis of the chloroplasts inside the leaves. The important findings of this study are the nanostructures on the surface of the hair-like fibers on the leaves of *O. aciphylla*, which not only have a water capture function but also reflect light. This has important theoretical significance for understanding how plant leaves in gravel deserts adapt to the environment.

## 1. Introduction

Water resources play a crucial role in the survival and reproduction of plants in arid regions [1,2]. In these areas, perennial plants employ various strategies to obtain the necessary water. Many plants primarily rely on their root systems to absorb water from the soil and supplement it by absorbing atmospheric moisture through their leaves, enabling them to thrive and reproduce successfully in these extreme environmental conditions [3,4,5,6,7,8]. Particularly in arid desert environments with sandy soils that are not conducive to retaining moisture, perennial plants can survive by relying on deep taproots to search for water sources several meters underground. Some plants have also evolved unique leaf structures, such as spines or glandular hairs, to capture atmospheric moisture or dew in high-temperature and arid conditions [9]. For example, in hot, dry, and low-rainfall sandy deserts, the prickly pear cactus (*Opuntia microdasys*) has specialized its leaves into spines and glandular hairs to capture airborne moisture [9], and its extensive root system can penetrate deep into the ground and spread around to access soil moisture [10]. In temperate sandy desert soils, plants like *Haloxylon ammodendron*, a perennial small shrub, have well-developed root systems with deep taproots that extend several meters into the ground [11], relying on groundwater for their water needs and physiological responses [12]. *Tamarix ramosissima* is another perennial tree with scale-like leaves and deep root systems that can extend over 3 m downwards and expand horizontally beyond 3 square meters, forming large thickets of Tamarix in sand dunes [13,14]. *Alhagi sparsifolia* is a perennial semi-shrub with roots that can quickly extend more than 10 m underground when the water table is deep, with its extensive roots searching for groundwater in a wide soil space [15]. The above-ground part of Alhagi sparsifolia is of relatively low height, with small ovate leaves that reduce water transpiration [16]. When the water table is shallower, its root system can also grow rapidly horizontally, expanding and producing suckers to generate new plant individuals to compete for light resources [17].

Despite the presence of well-developed root systems in plants growing in arid regions above sand dunes and rooted between sand seas, enabling them to effectively access groundwater, plants in temperate gravel desert environments often face greater challenges. These plants struggle to grow their roots both vertically and horizontally and often need to navigate around hard gravel layers to reach the water source in the soil beneath the gravel. Even when plants employ this strategy, the presence of gravel significantly restricts both vertical and horizontal root growth, making it difficult for plants in gravel deserts to access sufficient water, leading to more severe water stress.

This study focuses on a perennial, low-growing, cushion-like semi-shrub plant known as *Oxytropis aciphylla* Ledeb., which grows in gravel deserts (Gobi). Although *O. aciphylla* is of low stature and crown width [18], the presence of gravel in the soil limits the growth of its roots and the extension of its taproot. As a result, its root system is relatively underdeveloped. The presence of gravel also significantly limits the opportunity for *O. aciphylla* roots to access water from the soil beneath the gravel layer. This raises an important question: how does *O. aciphylla* obtain the necessary water in such a harsh environment? Through long-term field observations of *O. aciphylla*, we found that in the morning, the leaf surfaces of *O. aciphylla* are often moist, sparking our interest in the structural characteristics of the above-ground parts of this plant. Therefore, this paper aims to explore the mechanism behind this phenomenon, starting with the external structure of *O. aciphylla* leaves.

## 2. Results

### 2.1. Leaf Surface Morphological Features

**Figure 1:**
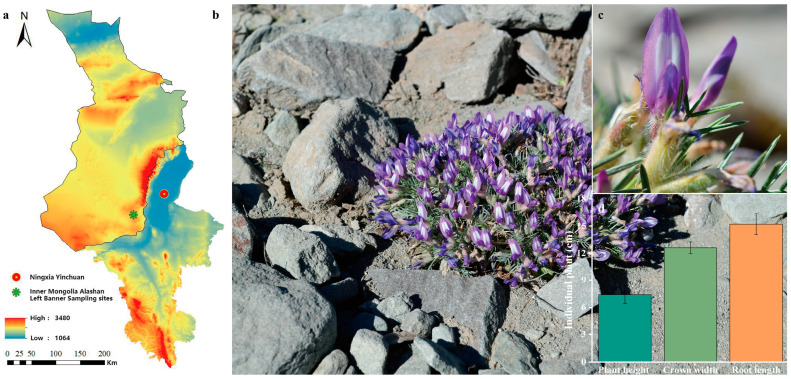
*O. aciphylla* growth in the gravel desert. (**a**). Sampling location of *O. aciphylla* in the southern part of Alashan Left Banner, which is adjacent to the western side of Helan Mountain in Ningxia Hui Autonomous Region, China; (**b**). Optical image of densely growing *O. aciphylla* plants in the field; (**c**). Purple flowers and curled leaves shown in the upper right corner of (**b**); (**d**). Measurement values of individual *O. aciphylla* parts.

**Figure 2:**
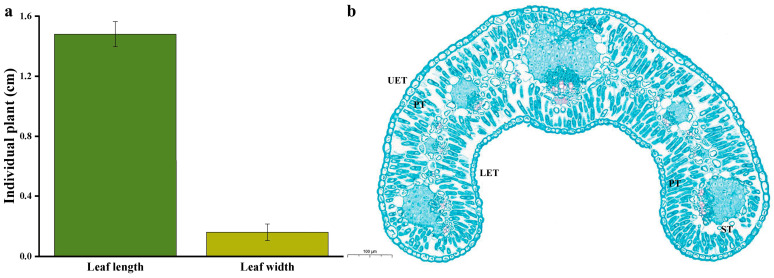
Various parameters of the leaves of *O. aciphylla*. (**a**). Measured values of individual leaves of *O. aciphylla*; (**b**). Cross-sectional image of an *O. aciphylla* leaf. UET represents upper epidermal cell thickness, PT represents palisade parenchyma thickness, and LET represents lower epidermal cell thickness. Spongy parenchyma (ST) is minimal.

**Figure 3:**
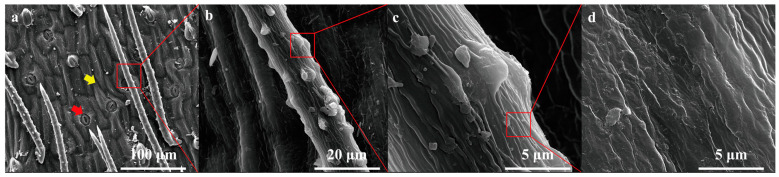
Scanning electron microscopy (SEM) images of an *O. aciphylla* leaf. (**a**). Surface structure of an *O. aciphylla* leaf (yellow arrows indicate large grooves on the surface, red arrows indicate stomata); (**b**). Structure of a single trichome on the leaf surface; (**c**). Structure of the raised surface on the trichome; (**d**). Structure of the non-raised surface on the trichome.

*O. aciphylla* is a plant that resides year-round in the arid desert Gobi region of northwestern China. The samples were collected in the southern part of Alashan Left Banner, Alxa League, Inner Mongolia, which is near the southwestern side of Helan Mountain in Ningxia. The area is characterized by a gravel desert terrain formed by the alluvial action of the Helan Mountain river (Figure 1a,b). *O. aciphylla* plants typically grow in clusters. Individual plant height measures 7.40 ± 0.96 cm (*n* = 5), crown width is 12.60 ± 0.65 cm (*n* = 5), and root length is 15.20 ± 1.15 cm (*n* = 5) (Figure 1d). The leaves of *O. aciphylla* are small and linear with a rolled appearance (Figure 1c), measuring 1.48 ± 0.08 cm in length (*n* = 5) and 0.16 ± 0.05 cm in width (*n* = 5) (Figure 2a). The apex of the leaf axis is woody and terminates in a sharp spine. The leaf epidermis is a structure composed of a single layer of cells, with stomata present on the surface of the epidermis. The leaf tissue differentiates into multilayered palisade tissue. The upper epidermal thickness (UET) is 14.69 ± 2.31 µm (*n* = 5), the lower epidermal thickness (LET) is 11.36 ± 1.92 µm (*n* = 5), the length of palisade tissue (PT) is 29.36 ± 2.94 µm (*n* = 5), and the width is 8.36 ± 0.85 µm (*n* = 5). Spongy parenchyma (SP) is minimal (Figure 2b). The leaf surface is densely covered with trichomes (Figure 3a), with each trichome measuring approximately 288.64 µm in length and 12.08 µm in width, with an apex angle of approximately 26.02°. The raised structure on the trichome surface is about 1.77 µm high, approximately 4.46 µm long at the base, with an angle of about 38.02° with the base (Figure 3c). The grooves on the trichome surface that is not raised are approximately 0.38 µm deep (Figure 3d).

### 2.2. The Mechanism of Water Collection by Leaf Trichomes

**Figure 4:**
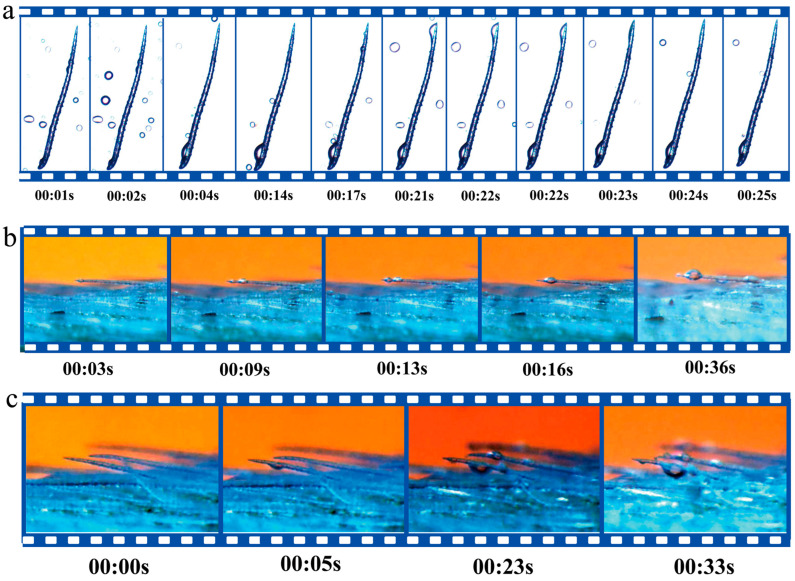
Water collection process of *O. aciphylla* leaf trichomes. (**a**). Water collection process of a single trichome on the leaf; (**b**). Water collection process of a single enlarged trichome on the leaf surface; (**c**). Water collection process of multiple trichomes on the leaf surface.

**Figure 5:**
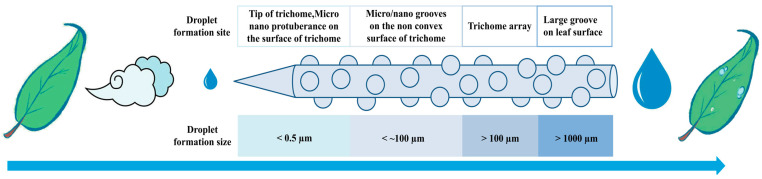
Schematic diagram of the water droplet capture mechanism by individual trichomes on the surface of *O. aciphylla* leaves (in the center of the image is an individual trichome on the leaf surface: conical, with multiple near-spherical protrusions on surface).

Water collection experiments were conducted to observe the trichomes on the leaves of *O. aciphylla*, and the results demonstrated that these trichomes play a role in both capturing and directing water droplets (Figure 4a–c). Individual trichomes on the leaves of *O. aciphylla* capture water droplets (Figure 4a), and the water collection process is magnified in specific regions of individual trichomes (Figure 4a,b). Furthermore, multiple trichomes on the leaves of *O. aciphylla* cooperate in the process of water collection (Figure 4c). The base of the trichomes on the leaf surface of *O. aciphylla* contains numerous stomata (Figure 3a). Water droplets are captured by the conical structure of individual trichomes (Figure 4a,b), and the Laplace pressure difference on the surface of the trichomes with micro-convex structures (Figure 5) drives the movement of liquid droplets from the tip to the base of the conical structures, where they coalesce in approximately 5 s, gradually forming larger droplets (Figure 4c). Adjacent individual trichomes create open capillary gaps for the collection of water. The collected water is funneled through the base of the trichomes and into larger droplets via the large grooves on the leaf surface, eventually entering the interior of the leaf through the leaf stomata. The water in the air captured by the trichomes is ultimately absorbed by the leaf. It takes approximately 4–5 s for droplets to form on the trichomes (Figure 4a–c). Thus, the leaves of *O. aciphylla* feature an array of multiple individual trichomes that cooperate in the water collection mechanism.

### 2.3. The Spectral Characteristics of the Leaves and Chloroplast Genome Features

**Figure 6:**
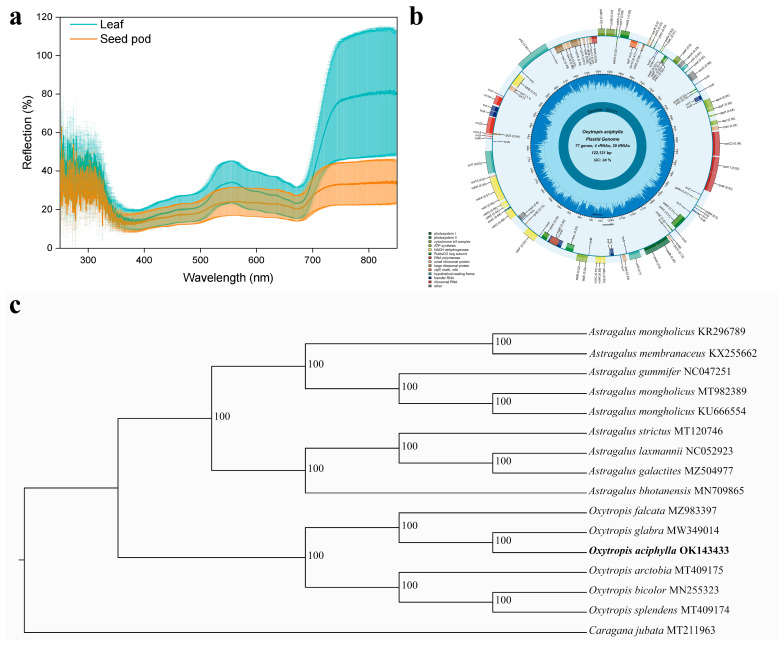
Response of *O. aciphylla* leaf surface to light rays and chloroplast genome features. (**a**). Reflectance spectra of *O. aciphylla* leaf and pods; (**b**). Chloroplast genome map of *O. aciphylla*; (**c**). Phylogenetic tree of chloroplast genomes in the Fabaceae family. The numbers to the right of the branches are bootstrap support values.

Through reflectance spectroscopy ranging from 200 nm to 850 nm on the leaves and pods of *O. aciphylla*, the results showed that the leaves reflected light rays in the spectral range of 200 nm to 320 nm, 550 nm, and 750 nm to 850 nm, while the pods also reflected light rays in the range of 200 nm to 320 nm and 740 nm to 850 nm. However, the leaves exhibited a relatively stronger reflection in comparison to the pods (Figure 6a).

The chloroplast genome (chloroplast DNA, cpDNA) of *O. aciphylla* had a length of 122,121 bp, comprising an 88,235 bp large single copy (LSC) region, a 10,400 bp small single copy (SSC) region, and a single 23,486 bp inverted repeat (IR) region. This genome contained 109 genes, including 76 protein-coding genes (PCGs), 4 rRNA genes, and 29 tRNA genes, with an overall GC content of 34.3%. Among these genes, those distributed in the LSC and SSC regions were associated with photosystem I (psa) and photosystem II (psb), including genes like psaA (0.5), psaB (0.55), psaC (0.69), psaJ (0.53), psaI (0.52), and psbA (0.53), psbB (−0.55), psbC (0.51), psbD (0.31), psbE (0.55), psbF (0.5), psbH (−0.33), psbI (0.73), psbJ (0.58), psbK (0.53), psbL (0.63), psbM (0.5), psbN (0.52), psbT (0.59), and psbZ (0.4) (Figure 6b).

A maximum likelihood (ML) tree was constructed using *Caragana jubata* as an outgroup (Figure 6c), which classified the entire tree into three main branches: *Oxytropis* with six species, *Astragalus* with nine species, and *Caragana* with one species. *O. aciphylla* shared the closest relationship with *Oxytropis glabra*.

## 3. Discussion

This study explores the structural features of the leaf surface of *O. aciphylla*, a plant growing in gravel deserts. We discovered that the densely arranged trichomes on the surface of *O. aciphylla* leaves exhibit elongated conical geometrical shapes with numerous nanoscale protrusions, while non-protrusion areas feature nanoscale grooves. These unique trichome structures enable *O. aciphylla* plants to efficiently capture moisture from the air. Furthermore, we observed that the *O. aciphylla* leaves exhibit a curled shape, and the trichome structures on the leaf surface can also reflect ultraviolet and near-infrared radiation. An analysis of the chloroplast genome of *O. aciphylla* leaves revealed the presence of numerous genes related to photosystem I and photosystem II, with no gene deletions. The densely arranged trichome structures on the leaf surface do not impede the plant’s photosynthesis process.

### 3.1. Dew as a Vital Water Source in Arid Ecosystems

In arid ecosystems, the scarcity of water resources is a major limiting factor affecting plant survival and growth [19]. Most sandy desert plants possess potential drought tolerance mechanisms and adaptation strategies, mainly relying on the growth of their primary root systems to access water from deep underground layers [20]. In gravel deserts, the near-surface soil has lower water content, making atmospheric dew one of the primary water sources for plants in this region. The adsorption and condensation of water are typical features of gravel desert ecosystems. Although the absolute amount of dew formation may not be high, it often exhibits a “high frequency, low yield” characteristic [21]. For instance, in semi-arid regions of China, daily average dew condensation on the soil surface covered with gravel was estimated to be 0.071 mm per day, with extreme dew yields ranging from 0.20 mm to 0.022 mm per day. However, compared to sandy and arid loess soils, gravel-covered soil surfaces experience reduced dew deposition, possibly due to the higher daytime temperatures (1–4 °C higher than soils covered with sand) [22,23]. Additionally, gravel acts as thermal insulation, helping to retain soil warmth during the night. Moreover, gravel exhibits slower heat dissipation from the afternoon to evening, resulting in higher soil temperatures, which inhibit air moisture condensation [23]. Therefore, plants in gravel deserts significantly increase their relative humidity, water potential, and morning photosynthetic performance by absorbing dew, thus mitigating the adverse effects of long-term drought.

Studies have shown that water condensation occurs when the relative air humidity (RH) exceeds a threshold of RH ≥ 30% [21], and air moisture condensation takes place at RH ≥ 35% in desert air [24]. For plants, light inhibition and general senescence may be related to the effects of long-term water stress under natural arid conditions. The supplementation of dew can potentially play a role in avoiding irreversible damage to the photosynthetic apparatus of plants when the relative water content (RWC) falls below 30% [25]. Dew, therefore, plays a crucial role in alleviating water deficiency and mitigating the adverse effects of long-term drought on plants. In arid ecosystems, in addition to rainfall, dew serves as an additional water source for plants. For example, many drought-resistant moss plants in deserts utilize their unique structural feature, namely trichome structures, to capture moisture from the air [26].

In arid and semi-arid regions, dew is a vital water source for plants. The leaves of *Combretum leprosum*, which grows in arid and semi-arid areas, utilize trichome structures to absorb dew [27]. It is also reported that plants growing in rocky or gravel deserts supplement their physiological water needs by utilizing atmospheric humidity. The leaves of *Croton*, which grow in highland rock crevices, have trichome protrusions that can absorb moisture from the unsaturated atmosphere to compensate for limited soil water supply [28]. *Reaumuria soongorica*, growing in gravel deserts similar to the red sand habitat, has fleshy, scale-like leaves with special valve microstructures, each featuring 4–7 inverted conical pores. These valves can contract and form an impermeable covering under low relative humidity, but under the influence of air humidity, they gradually expand and open the valves. These open inverted conical valves can absorb moisture from the atmosphere [29]. However, the specific dynamic process of water absorption by inverted conical valves has not been reported in the literature to date.

In a gravel desert similar to the habitat of red sand, this study discovered a widespread, highly drought-resistant cushion-like dwarf shrub, *O. aciphylla* Ledeb., whose leaf surfaces are covered with trichomes. These trichome structures can capture atmospheric moisture and reflect near-infrared radiation, aiding the plant’s adaptation to its environment. The ability of *O. aciphylla* leaves to capture atmospheric moisture is primarily attributed to the unique trichome structures on the leaf surface. Grooves around the leaf epidermis allow the collection of captured moisture and its entry into the leaf for plant absorption. Although there is ample evidence suggesting that plants growing in arid environments can acquire moisture from the air as an additional strategy to cope with and mitigate drought stress [30], this study is the first to demonstrate, through in situ experiments, that trichomes on *O. aciphylla* leaves can capture atmospheric moisture efficiently (Figure 4a–c).

### 3.2. Fiber Interface Structure as a Key for Moisture Transport

In nature, many fibers with directional fog collection capabilities have been reported and discovered. For example, spider webs often accumulate numerous small water droplets in the early morning, indicating their efficient ability to collect moisture from the humid air. Zheng et al. [31] discovered that spider silk can form periodic spindle-shaped structures composed of micro and nanoscale fibers. This multi-scale structure with nanometer- and micrometer-level features allows the gradient change in surface energy to drive directional droplet transport toward the spindle nodes, promoting continuous fog water collection activities. Inspired by spider silk, a series of biomimetic spider silk periodic spindle node fibers have been prepared using various methods [32], including solution coating, fluid coating, electrospinning, microfluidic techniques, and wet self-assembly. Bai et al. [33] used a solution coating method to produce fibers with periodic spindle node structures inspired by the instability of PMMA polymer solutions on nylon fibers. These fibers exhibit directional water-collection functions similar to spider silk. Bai et al. [34] further investigated the adhesion behavior between biomimetic spider silk structural fibers and droplets, theoretically explaining why spindle node structures on fibers can capture larger droplets compared to regular circular structures. Xue et al. [35] prepared spider silk-like periodic spindle node fibers with gradient size variations by controlling the drawing speed during fluid coating, achieving adjustable directional fog collection and transport. Feng et al. [36] used azobenzene polymers to prepare spider silk-like periodic spindle node fibers with micro and nanoscale structures. They controlled droplet aggregation and dispersion on spindle nodes using ultraviolet and visible light switching, achieving the manipulation of light-responsive tiny droplets. Du et al. [37] prepared a radial array of periodic spindle node structure biomimetic spider silk fibers using electrospinning. They efficiently collected water due to the capillary effect of spindle node structures and the Laplace pressure difference generated by the radial distribution of fiber angles. Chen et al. [38] revealed the microstructure of trichomes on certain plant surfaces, such as the high and low edges of the multiple-level micro-nano grooves of Sarracenia trichomes. This structure allows for two consecutive different transportation modes of liquid on the surface under dry and wet conditions, resulting in a “following waves push forward” water transport effect. The array of trichomes on *O. aciphylla* leaves is composed of multiple fibers with fog collection and dew functions. The individual trichome fibers have many micro-nano protrusions that are similar to the spindle nodes of spider silk, while the non-protruding structures on the trichomes are similar to the micro-nano groove structures of Sarracenia trichomes. The multi-level asymmetrical structure of the trichomes on *O. aciphylla* leaves has anisotropic properties. During the process of droplet condensation, droplets first randomly condense on the surface and gradually converge toward the tip, forming droplets at the tip. The droplets grow in size and move and collect in the direction of increasing the diameter of the conical trichome fiber, achieving directional transport of droplets from micro to macro scales. Capillary phenomena also occur between trichomes, accelerating the speed of liquid transport. This study found that the redistribution of droplets during the merging process is induced by the asymmetrical structure, leading to the tip effect. The results are similar to the functionality of 3D-printed conical arrays used by Feng et al. [39].

### 3.3. Fiber Interface Structure Can Generate Light Selection

Climate change can drive the evolution of unique structures in certain parts of organisms, providing competitive advantages for survival in harsh environments. Biological surfaces with special structural fibers can selectively affect solar radiation. For instance, polar bears (Ursus maritimus) that live year-round in the snow-covered and drifting ice regions near the Arctic Ocean have hollow-structured fur that can selectively absorb solar radiation heat without displaying any color [40,41]. In contrast, silver ants (Cataglyphis bombycina) that inhabit one of the hottest and driest environments on Earth, the Sahara Desert, have triangular silver hairs on their bodies that serve as a form of Mie scattering and full internal reflection, enabling them to scatter and reflect visible light and near-infrared radiation. This ability allows silver ants to forage at midday when their predators are heat-avoiding under extreme high temperatures [42]. Some plants have also evolved specialized reflective surfaces and insulating materials to isolate solar radiation heat during hot weather, minimizing leaf heat absorption. For example, the leaves of Populus tomentosa are covered on the bottom with a layer of hollow fiber-like structures. This layer reflects up to 55% of solar radiation, significantly reducing leaf heat absorption and protecting the leaves from scorching [43].

**Figure 7:**
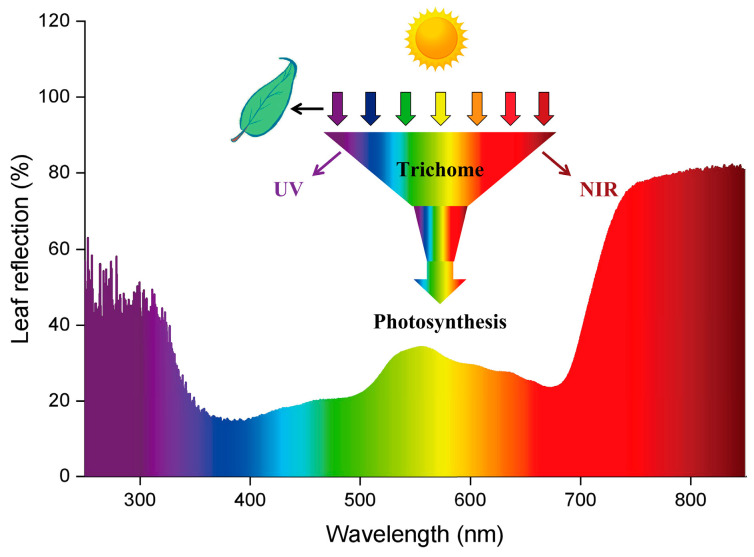
Schematic representation of trichome reflection of ultraviolet and near-infrared light on the *O. aciphylla* leaf surface. The densely covered trichomes on the leaf surface act as light funnels, filtering out ultraviolet and near-infrared light rays from sunlight and allowing only red and blue light to pass through the trichomes to reach the leaf cells for photosynthesis.

Plants surviving in arid environments employ various leaf survival strategies. The leaves of extreme arid desert plants have spines and glandular hairs. Arid desert plants typically have narrow linear leaves with trichomes, while semi-arid desert or desert shrub plants have small linear leaves with densely covered trichomes. For solar radiation, green plants selectively absorb the spectral range of visible light capable of photosynthesis [44]. Infrared light is absorbed by water within the plant body, generating heat and driving transpiration [45]. Radiations with wavelengths longer than 700 nm are strongly reflected and less absorbed by leaves [46]. In this study, we measured the reflection of light spectra from 200 nm to 850 nm by *O. aciphylla* leaves and found strong reflection in the ultraviolet and near-infrared regions. The leaves of *O. aciphylla* possess the ability to select sunlight efficiently. They reflect the near-infrared radiation that is intense and reduce water loss due to radiative heat (Figure 6a and Figure 7). Additionally, spectral testing of the trichome surface structure and the analysis of the chloroplast genome characteristics of the leaves showed that the densely arranged trichome structures on the leaf surface can reflect solar radiative heat without affecting photosynthesis (Figure 6b). Generally, many kinds of plants’ migrations are isolated in extremely arid desert or desert regions [47]. By constructing the chloroplast genome evolutionary tree of *O. aciphylla* and studying the phylogenetic relationship and population genetics of species in the spiny bean genus, it was found that *O. aciphylla* has the closest relationship with *O. glabra* (Figure 6c)*,* which also grows in the northwestern region of Xinjiang, China [48]. The leaves of *O. aciphylla* differ from those of *O. glabra* in both leaf surface structure and trichome characteristics [49]. Therefore, this close relationship is likely a result of geographical isolation and adaptive variations driven by the presence of gravel deserts in the region.

This study found that *O. aciphylla* leaves are typically curled, reducing the leaf’s exposure to solar radiation. Although plants growing in gravel deserts face higher temperatures and transpiration pressures than those growing in sandy deserts, the special structure of the fibers on *O. aciphylla* leaves efficiently captures atmospheric moisture and reflects solar radiation, aiding their adaptation to gravel desert environments. Moreover, the sharp spines and densely arranged trichomes on *O. aciphylla* leaves may serve a defensive function against herbivores, which requires further research in the future. Therefore, the trichome structures on the surface of *O. aciphylla* leaves not only capture atmospheric moisture but also reflect solar radiative heat, mitigating transpiration caused by radiative heat. This study reveals how plants adapt to extremely arid environments through their unique leaf morphologies and structures, providing valuable insights into adaptive strategies for organisms in different ecosystems. This research can be used in breeding drought-resistant plant varieties. Additionally, this research underscores the importance of understanding plant water acquisition and survival strategies in the context of climate change.

## 4. Materials and Methods

### 4.1. Leaf Collection

In July 2021, adult *O. aciphylla* plants were collected in the gravel desert (Gobi) area at the border of Yinchuan City, Ningxia Hui Autonomous Region, China, and the southeastern part of Alxa Left Banner, Inner Mongolia Autonomous Region, China (38°08′53.92″ N, 105°54′29.08″ E) for experimental testing. Leaf size and plant size, as well as the length of the root system, were measured using calipers and a centimeter ruler. The sample collection complied with the “Wild Plant Protection Regulations of the People’s Republic of China” (State Council Order No. 204, 30 September 1996). The specific coordinates of the sampling area were plotted using ArcGIS software ArcMap 10.8 (Esri, West Redlands, CA, USA).

### 4.2. Leaf Tissue Observation

Healthy adult leaves were selected, and leaf sections measuring 0.5 cm × 0.5 cm were quickly fixed in a formalin–ethanol–acetic acid mixture and subjected to vacuum pumping. Standard paraffin sectioning was performed, involving dehydration, transparency, wax impregnation, embedding, and sectioning with a Leica microtome (RM2235, Leica Microsystems, Wetzlar, Germany) at a thickness of 8 μm. The sections were then stained, dried, dewaxed, rehydrated, double-stained with fuchsin and green, and mounted with neutral gum.

### 4.3. Leaf Surface Characteristics

Healthy adult leaves were rinsed with ultrapure water (UPH-II-10T, Chengdu Ultratek Scientific Co., Ltd. Chengdu, China) twice for 5 min each. A series of gradient alcohol solutions (30%, 50%, 70%, 80%, 90%, 95%, 100%) were used for dehydration, with each step taking 10 min. The samples were gently adhered to a conductive adhesive, and gold coating was applied using an ion sputter coater (Hitachi E-1045, Tokyo, Japan) with a gold film thickness of approximately 20 nm. The leaf surface ultrastructure was observed using a scanning electron microscope (SEM, Inspect, FEI Company, Hillsboro, USA). The maximum magnification of SEM was 650,000 times, with an accelerating voltage of 0.530 kV and a resolution of 2.2 nm at 1 kV and 1.0 nm at 15 kV.

### 4.4. Leaf Structure Measurement

The anatomical features of the leaves were observed using an OLYMPUS BX60 microscope with a microscopic imaging system. Ten fields of view were randomly selected from 3 to 5 sections, and photographs were taken using microscopic imaging system software. ImageJ (https://imagej.nih.gov/ij/, accessed on 14 November 2023, Fiji.App (1.54f)) software was used to measure and analyze leaf anatomical structure-related parameters, including the length (SL), width (SW), aperture (SA), perimeter (SP), and area (SA) of stomata in each image, as well as leaf thickness (LT), upper epidermal cell thickness (UET), lower epidermal cell thickness (LET), midrib diameter (DM), and thickness of palisade parenchyma (PT) and spongy parenchyma (ST), with calculation of the ratio of PT/ST.

### 4.5. Leaf Water Collection Experiment

Leaves and trichomes on the leaves were carefully mounted on glass slides. Deionized water produced by the Milli-Q water system (Milli-Q Reference, Inc., Bedford, MA, USA) was introduced into an ultrasonic humidifier (Yadou YC-D204, Shanghai, China) to generate fog. Leaf water collection was studied under a saturated mist flow at approximately 20–30 mm/s, following methods described by Ju [9] and Bei [4].

### 4.6. Chloroplast Genome Structural Analysis and Phylogenetic Tree Construction

DNA samples were extracted from fresh O. aciphylla leaves according to the method described by Doyle (1987) [50]. After passing through processes such as DNA sample detection, library construction, library inspection, and sequencing, the qualified libraries were pooled to the flow cell based on effective concentration and the required amount of target data. Sequencing was carried out using the Illumina high-throughput sequencing platform NovaSeq 6000. The obtained Illumina raw sequences (6.01 Gb) were edited using the NGS QC Toolkit v 2.3.3. Contigs were obtained from high-quality reads using Bankevich et al. (2012) [51] and annotated with the plan software (Huang and Cronk, 2015) [52]. The contig was submitted to GenBank (Accession: OK143433), and the samples were stored in the Ecosystem Laboratory of Northern Minzu University (Sample voucher No: NMU00047) [18]. The chloroplast genome map of O. aciphylla was generated using R software 4.1.1 and Chloroplot software [53] and manually checked.

In the Fabaceae family, 14 chloroplast whole-genome sequence files that have been published in the GenBank database at the National Center for Biotechnology Information (NCBI, https://www.ncbi.nlm.nih.gov, accessed on 1 November 2021) were collected, and a phylogenetic tree was constructed using the IQTREE v1 RaxML method (Random Accelerated Maximum Likelihood) [48]. *Caragana jubata* was used as an outgroup, and the best-fitting model was determined by 1000 bootstrap replicates using the bootstrap method [54,55]. This tree was constructed to determine the phylogenetic position of *O. aciphylla* and its relationship with other species.

### 4.7. Data Statistics and Analysis

R v3.6.0 and Origin v2023b were used for data analysis and visualization.

## 5. Conclusions

This study revealed that in the gravel deserts during the night or early morning, the solitary trichomes on the leaves of *O. aciphylla* can capture atmospheric water droplets. These captured droplets move from the tip of the trichomes’ conical structures towards the base. Approximately every 5 s, a water droplet is collected, and adjacent individual trichomes form an open capillary gap to collect moisture again, with larger droplets forming approximately every 30 s. The aggregated large droplets are funneled through the grooves on the leaf surface into the leaf stomata and are absorbed by the leaf. Consequently, the array formed by multiple solitary trichomes and the trichomes on the leaf surface eventually constitutes the water-capturing mechanism of *O. aciphylla* leaves. Moreover, the trichomes on the leaf surface of *O. aciphylla* can selectively perform photosynthesis through blue and red light rays within sunlight. They can also reflect ultraviolet and near-infrared rays, reducing leaf surface temperature. Therefore, *O. aciphylla*, which can survive in gravel deserts, possesses feathery compound leaves. Its small, curled leaf surfaces, densely covered with trichomes, facilitate the rapid transport of water droplets to the leaf stomata, where they are absorbed by the leaves. Additionally, these trichomes reduce solar radiation heat emissions, thus minimizing water transpiration. The trichomes on the surfaces of *O. aciphylla* leaves have a special water-capturing and reflective system, attributed to their asymmetrical geometric shapes and micro-nano protrusions on their surfaces. Understanding how *O. aciphylla* adapts to water stress in the long-term survival in gravel deserts is significant. This research can be used in breeding drought-resistant plant varieties. The results of this study also provide valuable insights for the research on droplets’ continuous, long-distance, and rapid self-transportation, as well as the development of biomimetic technologies for daytime radiative cooling and the collection and concentration of droplets of various scales.

## Data Availability

The data is contained within the manuscript.
